# French Validation of the Social Dominance Orientation Scale_7_ (SDO_7_) by Ho et al. (2015): A Two-Dimensional Approach to Social Inequality Support

**DOI:** 10.5334/irsp.1075

**Published:** 2026-04-09

**Authors:** Sylvain Ferreira, Armelle Nugier

**Affiliations:** 1 Laboratoire de Psychologie Sociale et Cognitive, France

**Keywords:** Social Dominance Theory, Social Dominance Orientation, Dominance, Egalitarianism, Scale Validation

## Abstract

This pre-registered study presents the French-language validation of the Social Dominance Orientation scale_7_ (SDO_7_), originally developed by Ho et al. (2015). The SDO is a key variable in research on intergroup relations. Over the past two decades, a bidimensional conceptualization has emerged, distinguishing between SDO-Dominance (SDO-D) and SDO-Egalitarianism (SDO-E). These subdimensions enhance our understanding of the legitimizing myths that sustain social hierarchy. According to the original work of Ho et al. (2015), and across three independent samples (N = 1121), we found that a four-factor model (SDO-D; SDO-E; Pro-trait; Con-trait) provided the best fit to the data compared to three alternative models. Additionally, we examined the concurrent validity of the subscales using partial correlations. Results indicate that the subscales are differentially associated with attitudes and endorsement of ideological beliefs. Specifically, SDO-D is more strongly associated with attitudes and ideologies that directly oppress disadvantaged groups, whereas SDO-E is more closely linked to subtle legitimizing myths that justify social inequalities. A short version of the scale is provided in the supplementary materials.

## Introduction

The Social Dominance Orientation (SDO) scale is a key instrument for assessing individual and societal differences in the prioritization of social groups. In a world where prejudices profoundly shape intergroup relations, robust measures of SDO are essential. Accurately capturing how individuals perceive the structure of social hierarchies and justify inequalities requires instruments that reflect the complexity of these processes. Ensuring that these tools remain valid and adapted to specific cultural contexts, such as France, is crucial for producing reliable knowledge and addressing contemporary issues related to prejudice and social inequality. The SDO_7_ scale, developed by Ho et al. (2015), has contributed significantly to our understanding of power dynamics between groups. However, within the French context, while previous versions of this scale have been used (Duarte et al. 2004), an updated validation appears necessary. A French-language adaptation that carefully considers item wording is required to accurately capture the two key subdimensions of SDO: Dominance (SDO-D) and Egalitarianism (SDO-E).

### Social Dominance Theory (SDT)

According to Sidanius & Pratto (1999; see also Pratto et al. 2006), SDT posits that all complex human societies are structured around social hierarchies that shape intergroup relations. Dominant groups accumulate economic, political, and social advantages that reinforce their power, while subordinate groups have limited access to resources and opportunities. A central construct within this framework is Social Dominance Orientation, which reflects the extent to which individuals endorse or reject political attitudes and preferences that sustain or mitigate group-based inequalities, as well as their desire to maintain hierarchical social structures (Pratto et al. 1994). SDO is a key psychological variable for assessing individual and societal differences in intergroup prejudice. It strongly predicts prejudice and discriminatory behavior toward minority groups. It is also linked to beliefs that reinforce social hierarchies and justify inequality, such as political conservatism, meritocracy, and system justification. Within the framework of SDT, these behaviors and ideologies are conceptualized as legitimizing myths, defined as ‘attitudes, values, beliefs, stereotypes, and ideologies that provide moral and intellectual justification for the social practices that distribute social value within the social system’ (Sidanius & Pratto 1999, p. 45). Legitimizing myths fall into two categories: hierarchy-enhancing (HE) myths such as racism, which reinforce inequality, and hierarchy-attenuating (HA) myths such as feminism, which challenge social hierarchies. Endorsement of these myths is influenced by individuals’ levels of SDO: those high in SDO tend to support hierarchy-enhancing ideologies and reject hierarchy-attenuating beliefs, whereas those low in SDO show the opposite pattern. In recent years, research on SDO has evolved to distinguish between two primary subdimensions: Dominance (SDO-D) and Egalitarianism (SDO-E) (Ho et al. 2012, 2015; Jost & Thompson 2000). This distinction offers a more nuanced understanding of the psychological mechanisms through which individuals contribute to maintenance of social hierarchies, refining predictions regarding their endorsement of various hierarchy-justifying ideologies and attitudes.

### The Subdimensions of SDO: Dominance and Egalitarianism

Congruent with the evolution of the theory, the original SDO scale developed by Pratto et al. (1994) was introduced as a unidimensional construct predicting a wide range of sociopolitical attitudes. The scale consists of 16 items, including eight positively and directly worded items (e.g., ‘Superior groups should dominate inferior groups’) and eight negatively and indirectly worded items (e.g., ‘All groups should be given an equal chance in life’). Recent research, however, suggests that the SDO scale can also be conceptualized as having a bifactorial structure (Ho et al. 2012; Jost & Thompson 2000) comprising two subdimensions: SDO-Dominance (SDO-D) and SDO-Egalitarianism (SDO-E). According to Ho et al. (2012), a key difference between the two related dimensions lies in their degree of subtlety, with SDO-E being more subtle. Specifically, SDO-D reflects support for direct hierarchical dominance, characterized by aggressive behaviors toward minority groups and beliefs that justify such oppression. In contrast, SDO-E captures opposition to intergroup equality, maintained through ideologies that subtly legitimize social inequalities, such as meritocracy and the rejection of redistributive policies (Ho et al. 2012, 2015). These subdimensions differentially predict intergroup attitudes: SDO-D is associated with overtly oppressive attitudes, such as racism, torture, and forced assimilation (Larsson et al. 2012), whereas SDO-E is linked to the endorsement of system-justifying beliefs, such as the just world belief (Jost & Thompson 2000). However, some studies have failed to establish a clear empirical distinction between the two subdimensions (Guimond et al. 2003; Kugler et al. 2010), highlighting the need for further investigation. The original scale developed by Pratto et al. (1994; see also Sidanius & Pratto 1999) helped identify this bifactor structure in some studies (Duarte et al. 2004; Ho et al. 2012; Jost & Thompson 2000). However, it exhibited a significant methodological bias: items measuring SDO-D were predominantly framed positively, whereas those assessing SDO-E were negatively worded. This imbalance raised concerns about the theoretical validity of the distinction. To address this issue, Ho et al. (2015) revised the scale by balancing item formulations across subdimensions. Their findings reinforced the distinct roles of SDO-D and SDO-E, showing that SDO-D predicts support for the oppression of immigrants while SDO-E is linked to opposition to affirmative action policies. Validating the SDO_7_ scale in French would provide a more reliable tool for examining these psychological dynamics within a new cultural context.

### The Present Study

The present study aims to validate a French version of the SDO_7_ scale developed by Ho et al. (2015). To this end, we adopt a methodology similar to that used by the original authors. Across multiple samples, we test a model with a four-factor structure using confirmatory factor analyses. We then examine the concurrent validity of each subdimension while controlling for the other, using semi-partial correlations on variables closely related to those selected by Ho et al. (2015).

The first objective is to assess the factorial structure of the scale. SDO_7_ addresses the biases present in SDO_6_ by systematically balancing the positively and negatively worded items within each subdimension: SDO-D Pro-trait (e.g., ‘Some groups of people must be kept in their place’), SDO-D Con-trait (e.g., ‘Groups at the bottom are just as deserving as groups at the top’), SDO-E Pro-trait (e.g. ‘We should not push for group equality’), and SDO-E Con-trait (e.g. ‘We should work to give all groups an equal chance to succeed’).

**Figure 1:**
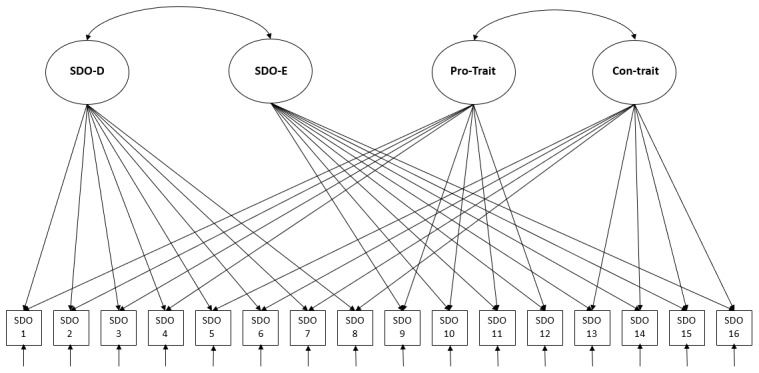
Four-factor confirmatory factor analysis model (SDO-D; SDO-E; Pro-trait; Con-trait).

While the scale is typically conceptualized as having a two-factor structure (SDO-D and SDO-E), it is important to examine the potential influence of item valence (Pro-trait vs Con-trait) (see Figure 1). Ho et al. (2015) validated a four-factor model; accordingly, we hypothesize that a four-factor model (SDO-D, SDO-E, Pro-trait, Con-trait) will provide a better fit than alternative models, including a two-factor model (SDO-D and SDO-E), a model based only on item formulation (Pro-trait and Con-trait), and a one-factor model based on an overall SDO score (H1).

The second objective is to assess the consistency of our translation with the French version of SDO_6_ developed by Duarte et al. (2004). Although that scale was validated based on the SDO_6_ version and took item wording into account, an updated French version aligned with SDO_7_ appears necessary, as the existing French translation is now over 20 years old. We expect our version to be strongly and positively related to the SDO_6_ scale by Duarte et al. (2004) (H2).

The third objective concerns the concurrent validity of the subdimensions. We selected scales similar to those used by Ho et al. (2015) in their validation, focusing on key constructs known to have robust associations with SDO. We anticipate that SDO-D will be strongly associated with attitudes and behaviors that promote the direct domination of low-status groups, such as prejudice against immigrants and sexual minorities, hostile sexism, support for hierarchy-enhancing policies (e.g., the death penalty), and attraction to hierarchy-enhancing professions (H3a). Conversely, SDO-E is expected to be linked to ideologies that subtly justify inequality, such as system justification and benevolent sexism, as well as opposition to redistributive social policies (e.g., affirmative action) and rejection of hierarchy-attenuating professions (H3b).

The fourth objective explores the relationship between SDO_7_ and personality traits. Based on Ho et al. (2015), we predict that SDO-D will be positively correlated with Neuroticism and negatively correlated with Agreeableness and Openness (H4a). Additionally, it is expected to be associated with traits from the Dark Triad (Machiavellianism, Narcissism, and Psychopathy; Paulhus & Williams 2002) (H4b). This research aims to deepen our understanding of the connections between SDO and personality.

## Method

### Translation of the SDO_7_ Scale

Items were translated from English to French, then back-translated by a bilingual researcher unfamiliar with the original scale. The two English versions were compared to ensure accuracy and conceptual equivalence, and any discrepancies were resolved. The French versions of the short and long scales are available in Appendices 1 and 2.

### Participants and Sample Size Determination

Following the recommendations of Rouquette & Fallissard (2011), we established a minimum threshold of 300 participants per sample to ensure sufficient statistical robustness. This threshold provides adequate representativeness and statistical power while considering resource constraints. However, to anticipate potential exclusions, we aimed to recruit up to 550 participants per sample.

For all three samples in this study, data were analyzed exclusively from individuals born in France. Sample 1 included 356 participants (*M* = 31.47; *SD* = 10.23; 46.4% men, 50.3% women, 2.2% other, 1% undisclosed). Sample 2 included 401 students (*M* = 21.89; *SD* = 5.33; 26.4% men, 68.6% women, 3.2% other, 1.8% undisclosed). Sample 3 included 364 students (*M* = 19.45; *SD* = 2.21; 9% men, 90% women, 1% other). Detailed demographic information is available in the supplementary materials (S1_Supplementary_Sample_Descriptives).

### Procedure

Participants completed a series of scales spontaneously, followed by demographic questions. They were then debriefed and thanked. The survey took approximately 15 minutes. The complete materials, data and analysis scripts and preregistration are available at the Open Science Framework: https://osf.io/ktyub.

### Measures

All scales used in this study are seven-point scales ranging from (1) ‘Strongly disagree’ to (7) ‘Strongly agree’, unless otherwise specified. To manage survey duration, different samples responded to specific scales, as indicated in parentheses. Scales were presented in a fixed order with the SDO_7_ scale always presented first. A detailed description of all scales is available in the supplementary analyses (S2_Supplementary_Analyses_Scale_Descriptives).

**The Social Dominance Orientation scale_7_** (Ho et al. 2015), translated into French (Samples 1, 2, and 3) (Appendix 1). This scale consists of 16 items measuring two subdimensions of SDO: SDO-D (Dominance) and SDO-E (Egalitarianism). After reversing the items concerned, a higher score indicates a tendency towards SDO-D and SDO-E (*S1*: α_ODS7_ = .92; α_ODS7-D_ = .85; α_ODS7-E_ = .89) (*S2*: α_ODS7_ = .89; α_ODS7-D_ = .79; α_ODS7-E_ = .85) (*S3*: α_ODS7_ = .87; α_ODS7-D_ = .78; α_7ODS-E_ = .83). See S3_Supplementary_Descriptives_SDO_7_ for details.

**The Social Dominance Orientation scale_6_**, Duarte et al. (2004) (Sample 1). This consists of 16 items and participants indicate their degree of agreement. A higher score reflects a strong inclination towards social dominance (α_ODS6_ = .92; α_ODS6-D_ = .86; α_ODS6-E_ = .89).

**The generalized prejudice against immigrants scale**, Dambrun & Guimond (2001) (Sample 1). This 15-item scale (e.g. ‘It is normal for illegal immigrants to be sent back to their country of origin’) assesses the level of prejudice against immigrants (α = .93).

**Support for the death penalty** (Samples 1 and 2). Support for the death penalty was assessed using a single-item measure (Dambrun 2007) in Sample 1 (e.g., ‘Are you in favor of the death penalty for a person convicted of a crime?’) and an eight-item scale (Sidanius et al. 2006) in Sample 2 (e.g., ‘We must have capital punishment for some crimes’, α = .95).

**Attitude towards affirmative action**, Kravitz & Platania (1993) (Samples 1 and 2). This scale includes six items (e.g., ‘affirmative action is a good policy’) translated into French for the purposes of the study, assessing attitudes towards affirmative action policies (*S1:* α = .91; *S2:* α = .90). A high score on these scales indicates high support for affirmative action.

**Occupational preferences** were measured using two four-item scales from Ho et al. (2015) (Samples 1 and 2), with responses ranging from 1 (‘Not at all attractive’) to 7 (‘Very attractive’). Participants rated the **attractiveness of hierarchy-enhancing jobs** (e.g., ‘criminal prosecutor’; S1: α = .81; *S2*: α = .83) **and hierarchy-attenuating jobs** (e.g., ‘public defender’; *S1*: α = .79; *S2*: α = .75).

**Ambivalent sexism scale**, Dardenne et al. (2006) (Sample 1). The 22-item scale assesses **hostile** sexism (11 items; e.g., ‘Most women interpret harmless remarks or actions as sexist’; α = .95) and **benevolent** sexism (11 items; e.g., ‘Women should be protected and loved by men’; α = .88), with higher scores reflecting greater sexism.

**System justification scale**, Labarre & Felonneau (2022; Kay & Jost 2003) (Samples 1 and 2). The scale is composed of eight items (e.g., ‘In general, the French political system works as it should’). A higher score indicates a greater tendency to perceive existing social, economic, and political systems as fair and legitimate—that is, a higher level of system justification. (*S1:* α = .84; *S2:* α = .78).

**Support for the Black Lives Matter (BLM) movement**, Holt & Sweitzer (2020) (Sample 2). This six-item scale (e.g., ‘In my opinion, the Black Lives Matter movement is’) assesses individuals’ support for the movement (α = .95).

**Modern Homonegativity Scale**, Morrison & Morrison (2003) (Sample 2). This 12-item scale assesses feelings of homonegativity towards homosexual people (e.g., ‘Many gays and lesbians use their sexual orientation so that they can obtain special privileges’) (α = .93).

**Big Five**, Plaisant et al. (2010) (Sample 3). This scale measures personality traits according to five factors—Openness, α = .75; Conscientiousness, α = .82; Extraversion, α = .81; Agreeableness, α = .72; Neuroticism, α = .83—with a total of 45 items (e.g., ‘I see myself as someone who is forgiving by nature’).

**Short Dark Triad**, French et al. (2022) (Sample 3). This 27-item scale (e.g., ‘Most people can be manipulated’) assesses three personality traits: Machiavellianism (α = .73), Narcissism (α = .67), and Psychopathy (α = .72).

**Social Desirability**, Crowne & Marlowe (1960) (Sample 3). This 13-item scale evaluates the tendency to respond in socially acceptable ways, with responses of ‘true’ or ‘false’ (kr20 = .52).

**Political measurement** (Samples 1, 2, and 3). Political orientation was assessed using two measures. First, political sensitivity was measured using a single-item nine-point scale ranging from (1): ‘Far left’ to (9): ‘Far right’. Second, political conservatism was assessed using two items rated on a seven-point scale evaluating economic and social attitudes. For economic political conservatism, a lower average score indicated more liberal views (i.e., in favor of minimal state intervention), whereas for social political conservatism, a higher average score indicated more conservative views (Sidanius et al. 2008).

**Demographic data**. The following demographic measures were included: gender, age, sexual orientation, country of birth of the participants and their parents, religious beliefs.

## Results

### Factor Structure of the SDO_7_

To address our first hypothesis (H1), we analyzed the factor structure of our scale. Based on Ho et al. (2015), we hypothesized that a four-factor model (i.e. SDO-D; SDO-E; Pro-trait; Con-trait) would better predict the data than alternative models, including a two-factor model based only on the subdimensions, a two-factor model considering only a two-factor model considering only the items wording, and a one-factor model capturing the general concept of SDO. As preliminary analyses revealed violations of multivariate normality, we used the MLR (robust maximum likelihood) estimator (Finney & DiStefano 2006).

To evaluate the goodness of fit of the tested models we used several indices (Tanaka 1993): the chi-square test statistic (χ^2^), the comparative fit index (CFI), the root mean square error of approximation (RMSEA), and the standardized root mean square residual (SRMR). A satisfactory fit is indicated by a CFI ≥ 0.90, an RMSEA ≤ 0.08, and an SRMR ≤ 0.08. In line with our expectations and the findings of Ho et al. (2015), the four-factor model provides a better fit to the data compared to the other models.^1^ Across all samples, this model yielded excellent fit indices (CFI = .973, TLI = .961, RMSEA = .046, SRMR = .046, χ²/df = 2.88). Moreover, comparisons between the four-factor model and each of the other three models indicated that the four-factor model was a significantly better fit in every case. Fit indices for each sample are presented in Table 1.

**Table 1:** Fit indices for a model with four factors, two-dimensional factors, two methodological factors, and one factor of the SDO_7_ scale with 16 items.


SAMPLE	MODEL	CFI	RMSEA	SRMR	χ²	df	χ²/df	χ²_difference_ test		

								χ²_difference_	*df*	*p*

**Sample 1**	One factor	.844	.111	.065	416.052	104	4.00	149.95	22	<.001

	Two factors (D vs E)	.891	.094	.060	323.941	103	3.14	99.95	21	<.001

	Two factors (Pro- vs Con-traits)	.872	.101	.060	359.405	103	3.49	117.19	21	<.001

	Four factors	.965	.059	.034	173.799	82	2.44	–	–	–

**Sample 2**	One factor	.814	.103	.071	428.868	104	4.12	71.18	22	<.001

	Two factors (D vs E)	.883	.083	.065	308.260	103	2.99	40.57	21	<.001

	Two factors (Pro- vs Con-traits)	.852	.093	.067	363.443	103	3.54	53.38	21	<.001

	Four factors	.931	.071	.036	856.641	82	10.44	–	–	–

**Sample 3**	One factor	.761	.112	.076	439.406	104	4.23	221.54	22	<.001

	Two factors (D vs E)	.851	.088	.067	326.116	103	3.17	146.78	21	<.001

	Two factors (Pro- vs Con-traits)	.801	.102	.072	383.525	103	3.72	185.51	21	<.001

	Four factors	.971	.043	.035	130.821	82	1.60	–	–	–

**Samples 1, 2 & 3** 2	One factor	.815	.107	.066	1032.828	104	9.93	556.66	22	<.001

	Two factors (D vs E)	.878	.087	.059	717.856	103	6.97	343.27	21	<.001

	Two factors (Pro- vs Con-traits)	.852	.096	.061	849.197	103	8.24	433.78	21	<.001

	Four factors	.973	.046	.046	236.337	82	2.88	–	–	–


*Note*. CFI: Comparative Fit Index, RMSEA: Root Mean Square Error of Approximation, SRMR: Standardized Root Mean Residual.

### Relations Between the Whole SDO_7_, SDO_6_, and Criterion Variables

To assess the concurrent validity of the overall SDO_7_ scale and to examine whether it meaningfully relates to the SDO_6_ scale and relevant external outcomes, we performed correlational analyses (Table 2). In developing the SDO_7_, Ho et al. (2015) not only addressed the imbalance in Pro- versus Con-trait wording of the -D and -E items in the SDO_6_ scale, but also revised items that could be interpreted as implying a preference for ingroup dominance rather than support for dominance hierarchies in general, thereby aligning the scale more closely with the conceptualization of SDO (Ho et al. 2015, p. 1004–1005). Our translation faithfully reflects these changes. In Sample 1, SDO_7_ was highly correlated with SDO_6_ (*r* = .93, *p* < .001). We also observed that SDO_7_-D was highly correlated with SDO_6_-D (*r* = .83, *p* < .001) and that SDO_7_-E was highly correlated with SDO_6_-E (*r* = .89, *p* < .001). These values are very similar to those found in Ho et al. (2015). As in Ho et al. (2015), SDO_7_ demonstrated relationships with all criterion variables that are consistent in both direction and magnitude with those observed for SDO_6_, thereby confirming our hypothesis (H2). As shown in Table 2, these relationships are generalizable across the three samples and are theoretically consistent with findings reported in the literature. Specifically, SDO_7_ is positively related to prejudice toward minorities (i.e., immigrants, homosexual persons, women), support for harsh punishment (i.e., death penalty), endorsement of hierarchy based on power and status (i.e. preference for hierarchy-enhancing jobs), political conservatism, and system justification. Conversely, SDO_7_ is negatively related with subtle hierarchy-enhancing ideologies and beliefs (i.e., preference for hierarchy-enhancing jobs, support for hierarchy-attenuating social policies such as affirmative action, support for the BLM movement, and economic political liberalism).

**Table 2:** Correlations and part-correlations between the SDO-D_7_, SDO-E_7_, SDO_7_, and SDO_6_ scales and the other variables.


	SDO-D	SDO-E	Part *r* difference	Correlation with	
			
	PART *r*	PART *r*	*t*	SDO_7_	SDO_6_

**Sample 1**					

*Variables primarily related to SDO-D*					

Prejudice against immigrants	.21***	.17***	1.14	.67***	.66***

*Death penalty support	.13*	.04	2.51**	.35***	.30***

*Hierarchy-enhancing jobs	.12*	.02	2.89**	.31***	.30***

Hostile sexism	.10^t^	.21***	–3.10**	.59***	.59***

*Variables primarily related to SDO-E*					

Benevolent sexism	.14**	.07	2.08*	.43***	.43***

*Hierarchy-attenuating jobs	.02	–.10*	3.41***	–.19*	–.19*

Support for affirmative action	–.08	–.08	0.04	–.34***	–.33***

System justification	.08	.08	0.07	.33***	.33***

*Economic liberalism	–.08	–.12**	1.11	–.43***	–.40***

Social political conservatism	.14**	.18***	–1.13	.59***	.58***

*Political conservatism	.12*	.19***	–2.09*	.60***	.59***

**Sample 2**					

*Variables primarily related to SDO-D*					

Homonegativity	.24***	.24***	0.08	.66***	–

Death penalty support	.17***	.12*	1.22	.45***	–

*Hierarchy-enhancing jobs	.10*	.07	0.64	.29***	–

*Variables primarily related to SDO-E*					

Hierarchy-attenuating jobs	–.06	–.01	1.32	–.14	–

*Support for affirmative action	–.08	–.11*	0.94	–.32***	–

*Support for the BLM movement	–.11*	–.29***	4.64***	–.58***	–

*System justification	.11*	.18***	–1.73^t^	.45***	–

Economic liberalism	–.10*	–.09^t^	–0.11	–.32***	–

Social political conservatism	.23***	.23***	0.002	.63***	–

Political conservatism	.25***	.21***	1.11	.64***	–

**Sample 3**					

*Variables primarily related to SDO-D*					

*Openness	–.12*	–.01	–2.38*	–.20*	–

*Agreeableness	–.14**	–.05	–1.84^t^	–.28***	–

Neuroticism	–.01	–.12*	2.33*	–.18^t^	–

Machiavellianism	.08	.15**	–1.34	.32***	–

Narcissism	.03	.12*	–1.89^t^	.23**	–

Psychopathy	.07	.14**	–1.42	.30***	–

*Variables primarily related to SDO-E*					

Extraversion	–.02	.06	–1.87^t^	.04	–

Conscientiousness	.006	–.04	.99	–.05	–

Economic liberalism	–.07	–.05	–0.50	–.18^t^	–

Social political conservatism	.19***	.14**	1.09	.45***	–

Political conservatism	.23***	.18**	0.98	.53***	–


*Note*: t *p* < .10. *p <.05. **p < .01. ***p < .001. Part *r* = semi-partial correlations. Variables for which the observed pattern aligns with our predictions (either because the *r* part difference is significant or because the variable is linked with one dimension only on the excepted direction) are marked with an asterisk (*).

To assess the incremental validity (ΔR²) of SDO_7_ over SDO_6_, we conducted hierarchical linear regressions for each criterion in Sample 1 (see details on OSF). In the first step, we entered SDO_6_ (overall score), and in the second step we added SDO_7_ (overall score). The results showed that, despite high multicollinearity between the two measures, the SDO_7_ overall scores accounted for additional variance (i.e., significant ΔR² changes ranging from .01 to .034, corresponding to small-to-medium effects according to Cohen’s (1988) guidelines) in several key criteria (e.g., prejudice, death penalty support, hostile sexism, support for affirmative action, economical political liberalism, social and political conservatism) beyond that explained by SDO_6_. Overall, our pattern of results provides converging evidence that SDO_7_ is the preferable measure for predicting hierarchy-relevant outcomes while nonetheless maintaining the well-established validity of the SDO_6_ scale.

### Concurrent Validity of the SDO_7_ Scale Subdimensions

We then focused on the SDO subdimensions. To assess the concurrent validity of the SDO-D and SDO-E subdimensions, we computed a series of semi-partial correlations with the criterion variables, controlling for the overlap between SDO-D and SDO-E (Table 2). This approach allowed us to determine whether the association between SDO-D and a given criterion variable was stronger than that between SDO-E and the same variable, after accounting for their shared variance.

In Sample 1, SDO-D emerged as more strongly associated with support for the death penalty, and with the perceived attractiveness of hierarchy-enhancing jobs. Conversely and in line with our expectation, SDO-E was more strongly and negatively associated with the attractiveness of hierarchy-attenuating jobs and was more strongly and positively associated with right-wing political orientation. Economic liberalism was negatively associated with SDO-E but was not associated with SDO-D. These findings support hypotheses H3a and H3b. However, the remaining results did not support our hypotheses. Specifically, unexpected associations were observed between SDO-D and benevolent sexism, and between SDO-E and hostile sexism. All other variables in this sample were similarly associated with both SDO-D and SDO-E.^3^ For this sample, we tested and found support for the invariance hypothesis (Sidanius & Pratto 1999), according to which men exhibit higher levels of SDO_7_ compared to women (S9_Supplementary_Men_Women_Comparison).

In Sample 2, SDO-D was slightly more associated with death penalty support than SDO-E and with the perceived attractiveness of hierarchy-enhancing jobs, which was not associated with SDO-E. However, the semi-partial correlations differences for these comparisons were not significant. As expected, SDO-E was more strongly and negatively associated with support for the BLM movement and showed a marginally and negatively stronger association with system justification. Support for affirmative action was significantly and negatively associated with SDO-E, but was not associated with SDO-D. These findings provide support for hypotheses H3a and H3b, whereas the remaining results did not align with our hypotheses. Indeed, the perceived attractiveness of hierarchy-attenuating jobs was not associated with either of the two subdimensions and the remaining variables in this sample showed similar associations with SDO-D and SDO-E.^4^

In Sample 3, we examined the relationships between SDO_7_, its subdimensions, and personality traits. The results indicated that SDO_7_ was negatively correlated with Openness, Agreeableness, and Neuroticism, yet marginally for this last variable. It was not associated with the two other traits of the Big Five (i.e., Extraversion, Conscientiousness). Furthermore, SDO_7_ was positively associated with all three dimensions of the Dark Triad: Machiavellianism, Narcissism, and Psychopathy. These results are consistent with those found in the literature and in Ho et al. (2015). Hypothesis H4a was partially confirmed. As expected, semi-partial correlations revealed that SDO-D was negatively associated with Openness and Agreeableness. However, contrary to expectations, SDO-E showed a stronger negative association with Neuroticism. No significant relationships were found between SDO-D, SDO-E, and the other Big Five traits. With respect to the Dark Triad and hypothesis H4b, the results unexpectedly indicated that SDO-E was more strongly associated with Machiavellianism, Narcissism, and Psychopathy.

### Discriminant Validity

To assess the discriminant validity of our scale, we examined its relationship with social desirability, expecting no significant association (Sample 3). Social desirability showed no meaningful relation with SDO_7_ (*r* = –.10), SDO-D (*spr* = –.08), or SDO-E (*spr* = .02), all *p*s > .10. These findings support the discriminant validity of our scale.

## Discussion

This study provides a French validation of the SDO_7_ scale (Ho et al. 2015). We first examined the factorial structure using a four-factor model that accounts for the subdimensions of SDO while addressing a key methodological limitation of early versions. SDO_7_ was mainly designed to correct the item valence bias observed in SDO_6_ by including both positively (i.e., Pro-trait) and negatively (i.e., Con-trait) worded items within each subdimension. This resulted in a four-factor structure: Dominance and Egalitarianism, which represent the two theoretical subdimensions, and Pro-trait and Con-trait, which capture item valence effects. Confirmatory factor analyses across three independent samples consistently showed that the four-factor model provided the best fit to the data, confirming the theoretical and methodological improvements introduced by SDO_7_. Beyond methodological refinement, the four-factor model also strengthens the theoretical validity of a two-dimensional SDO by demonstrating that the distinction between SDO-D and SDO-E is not merely a product of item wording but reflects two genuinely distinct psychological components of the construct.

These results validate the scale’s structure and suggest that the methodological bias identified in previous versions has been effectively addressed. Nonetheless, in practice, researchers typically compute only the overall scores for SDO-D and SDO-E. A short version of the scale consisting of eight items also demonstrated robust psychometric properties, making it particularly useful for studies that require brief measures.

### Subdimensions of SDO_7_ and Personality Traits

We further explored how SDO_7_ and its subdimensions relate to personality traits. Our findings align with existing literature, showing that SDO_7_ is negatively associated with Openness and Agreeableness (Hodson et al. 2009). Semi-partial correlations revealed that SDO-D primarily drives these negative associations. Regarding the Dark Triad, our results confirmed positive correlations between SDO_7_ and Machiavellianism, Narcissism, and Psychopathy (Hodson et al. 2009). Interestingly, SDO-E showed stronger associations with Dark Triad traits—an observation that diverges from Ho et al. (2015), who found stronger associations with SDO-D. These discrepancies point to possible cultural or contextual differences and suggest that personality traits may interact differently with each subdimension, offering a more nuanced understanding of the psychological basis of Social Dominance Orientation.

### Concurrent Validity

To assess the validity of our scale, we compared it with the current French version of the SDO_6_ scale (Duarte et al. 2004). Our findings show high consistency in the direction and magnitude of correlations across key variables, confirming the conceptual equivalence between the two versions. This indicates that the linguistic and structural adjustments made in SDO_7_ did not compromise its concurrent validity. On the contrary, additional analyses showed that SDO_7_ provides meaningful incremental validity beyond SDO_6_, supporting its use in contemporary research. As expected, SDO_7_ was positively associated with hierarchy-maintaining constructs and negatively correlated with egalitarian attitudes, which is consistent with previous work (Ho et al. 2012, 2015; Pratto et al. 1994; Sidanius & Pratto 1999). Finally, the absence of associations with social desirability further supports discriminant validity.

A central aim was to examine whether SDO-D and SDO-E are differentially associated with various attitudes and ideologies. Our findings provide some evidence for the two-dimensional conceptualization of SDO, with semi-partial correlations revealing distinct associative patterns between each subdimension and a broad range of social, political, and ideological attitudes. Specifically, SDO-D showed the strongest association with conservative and punitive attitudes, such as support for the death penalty, reflecting its alignment with ideologies that explicitly endorse hierarchy. In contrast, SDO-E was more strongly associated with opposition to social justice initiatives such as affirmative action and the BLM movement. These findings reinforce the value of distinguishing between the two subdimensions: while both reflect generalized support for inequality, they appear linked to distinct psychological mechanisms—direct endorsement of dominance versus subtle resistance to equality—offering a more nuanced understanding of how group-based hierarchies are maintained and justified. In this way, the present study contributes to a more refined conceptualization and application of the SDO framework in the study of social and political attitudes.

Beyond methodological and psychometric considerations, these findings should also be interpreted considering the French cultural context, which is characterized by strong egalitarian ideals and republican universalism (Guimond et al. 2014). In France, social norms strongly value equality and discourage explicit endorsement of group-based hierarchy. This cultural context may promote the rejection of overt dominance (lower SDO-D) while simultaneously allowing more subtle forms of hierarchy justification, such as meritocratic ideology (higher SDO-E). This may partly account for the relatively low mean levels of SDO observed in our samples compared with other validations. For instance, Ho et al. (2015) reported an average SDO score of 2.6 across six US samples, Carvalho et al. (2025) reported 2.61 for a Portuguese sample, and Aiello et al. (2019) reported 2.4 for an Italian sample—all on a seven-point scale—compared to an average of 2.2 across our three samples.^5^ Nevertheless, these values are close to those observed by Duarte et al. (2004), who reported mean SDO_6_ scores of 2.18 among French psychology students, and by Lankester & Alexopoulos (2023), who reported mean SDO_7_ scores of 2.29 (Study 1) and 2.47 (Study 2) in general French population samples.

While Ho et al. (2015) did not provide item-level means for the SDO_7_, preventing a direct comparison with our data, such a comparison is possible with the Italian validation by Aiello et al. (2019). Item-level distributions in the Italian sample are similar to those observed in our data, except for a few Dominance-related items (i.e., items 7 and 8) that show noticeably higher endorsement in the Italian dataset. In the Portuguese validation by Carvalho et al. (2025), the scores for most SDO-D items were even higher (*M* = 2.88). These differences are concentrated in statements referring to overt group-based dominance, suggesting that explicit hierarchical attitudes may be expressed more openly in the Italian and Portuguese contexts than in France. This pattern is consistent with the idea that cultural and normative contexts shape the acceptability of dominance-oriented expressions of hierarchy support, with French respondents tending to avoid explicit dominance statements while potentially maintaining more subtle hierarchy-justifying beliefs.

This interpretation also aligns with Ho et al.’s (2012) demonstration that the relative influence of SDO-D and SDO-E varies across the sociopolitical context, particularly as a function of the degree of conflict and instability in intergroup relations. In highly conflictual and hierarchical contexts, SDO-D more strongly predicts support for ideologies that explicitly legitimize domination. In contrast, in stable or normatively egalitarian societies—where intergroup conflict is less overt—SDO-E tends to be a stronger predictor of attitudes that subtly maintain hierarchy, such as opposition to redistributive policies or endorsement of meritocratic beliefs. France, as a Western democracy whose republican values convey strong egalitarian norms, represents precisely the type of sociopolitical context in which SDO-E should theoretically exert greater influence than SDO-D.

### The Two-Dimensionality of the SDO: A Robust Distinction?

Our findings invite further reflection on the empirical distinction between the two subdimensions of SDO_7_. Indeed, some attitudes and ideologies were not more effectively predicted by either SDO-D or SDO-E. First, prejudice toward immigrants showed comparable associations with both versions of the scale. We had initially hypothesized that SDO-D would be more strongly correlated with these prejudices, as they may reflect relatively explicit expressions of intergroup rejection. However, the similar association observed between the two subdimensions can be explained by the content of the prejudice scale items themselves, which simultaneously invoke a logic of intergroup hierarchy (e.g., ‘We should more strictly limit the entry of foreign families into France’) and opposition to the equalization of rights and resources (e.g., ‘French citizens should have priority in housing’). Thus, in our study, the rejection of immigrants appears to rest concurrently on both justification of social hierarchy and opposition to equality, which makes the similar association observed with both dimensions coherent. Second, our hypotheses regarding sexism were not confirmed in the expected direction. We had anticipated that hostile sexism, given its direct and oppressive nature, would be more strongly associated with SDO-D, whereas benevolent sexism, given its more subtle character, would be more closely related to SDO-E. However, the results reveal the opposite pattern. This finding can nonetheless be interpreted in light of the work of Dardenne et al. (2006), who suggest that benevolent sexism functions as an ideology justifying male dominance rather than as an egalitarian position. As these authors emphasize, a ‘benevolent sexist attitude would function as an ideology justifying the dominance of the group of men over that of women and not as an egalitarian ideology’ (Dardenne et al. 2006, p. 258). Conversely, hostile sexism may today function more as a reaction to egalitarian claims, particularly feminist ones, which would explain its stronger association with SDO-E. In Dardenne et al. (2006), SDO-E, measured with the SDO_6_, was not related to hostile sexism. It is possible that this absence of relationship may be attributable to the psychometric properties of the earlier version of the SDO scale.

Berry (2022), in his meta-analysis, examines studies that support a distinction between the two subdimensions of the SDO using the SDO_7_ scale. He argues that the distinction between SDO-D and SDO-E is weakly supported by the data for several reasons. First, the meta-analytic correlation coefficient between the two subdimensions is very high, raising concerns about the independence of the two constructs. Second, SDO-D and SDO-E exhibit very similar correlation magnitudes with a variety of variables, suggesting that the two subdimensions may not capture fundamentally different constructs. Third, the means and standard deviations of SDO-D and SDO-E are almost identical across the studies examined. Together, these findings cast doubt on the empirical robustness of the two-dimensional model. Both our results and those of Berry (2022) highlight some ambiguity regarding the two-dimensional structure of SDO. Although the theoretical distinction between SDO-D and SDO-E is well established, empirical data do not always strongly support this separation. Two interpretations may account for this: either SDO is fundamentally a unidimensional construct, or the current SDO_7_ scale does not adequately capture the theoretical nuances between the two subdimensions. The latter hypothesis appears more plausible. Despite some inconsistencies, certain attitudes and ideologies were more strongly predicted by one subdimension than the other. Indeed, our findings and those of other researchers (Ho et al. 2012, 2015; see also Aiello et al. 2019; Carvalho et al. 2025) provide evidence in favor of a two-dimensional structure.

### Recommendations for Use

The French version of the SDO_7_ scale validated in this study provides a reliable and theoretically coherent tool for measuring SDO. The findings obtained with the SDO_6_ remain valuable and noteworthy and new research conducted with the SDO_7_ should be viewed as a continuation and refinement of the work initiated with the SDO_6_ and the previous versions of the SDO scales (see Ho et al. 2015). Nonetheless, if researchers aim to compare subdimensions to better understand the distinct routes that contribute to supporting hierarchical structures in the world and their consequences, it is preferable to use the new SDO_7_ scale as it addresses key methodological limitations of earlier measures.

The SDO_7_ scale offers several improvements over previous versions. First, it provides robust psychometric properties. Second, it provides better control over item wording effects (Pro- vs Con-trait items), thereby enhancing both theoretical and methodological validity. Third, it clarifies the underlying nature of the dominance dimension by reformulating items that could be misconstrued as mere ingroup favoritism rather than generalized support for group-based hierarchy (Ho et al. 2015). Fourth, it provides robust psychometric properties and enables examination of item valence effects. We therefore recommend that SDO_7_ be the preferred choice when researchers aim to (a) empirically test the bidimensional structure of SDO, (b) analyze the two subdimensions separately to examine their differential predictive validity, and (c) conduct cross-national comparisons, as SDO_7_ is now the most widely used version in the international literature (Aiello et al. 2019; Ho et al. 2015).

The use of the scale should depend on the interest and hypotheses of the researchers. If researchers are interested in the general relation between SDO and other psychological concepts, and/or if they do not have specific hypotheses on the motivation (dominance or anti-egalitarianism) underlying intergroup attitudes, the better option is to rely on the total score of the scale, which captures overall preference for group-based inequality. However, in contexts where deeper exploration of the psychological motivations behind intergroup attitudes is required, researchers should prioritize models that account for the differential predictive validity of these two subdimensions in introducing each dimension as a covariate for the other. We recommend using the *full* 16-item version of the scale whenever feasible and reporting both subdimension scores, as this preserves the conceptual distinction between overt dominance and opposition to equality.

### Limitations and Perspectives

This study has several limitations that should be acknowledged. First, as a correlational study, it identifies associations between variables but does not allow for causal inferences. Future research using longitudinal or experimental designs would help clarify the directionality of these relationships. Second, differences between our findings and those reported by Ho et al. (2015) may be partly attributable to variations in measurement instruments and to the measurement procedure. Several of the scales used in our study were adapted from English, which may have introduced issues of translation accuracy or cultural equivalence that could have impacted reliability and validity. The presentation of the scales was not randomized, which may have introduced order effects or artificially increased correlations between certain constructs. However, as the study focused on stable constructs and did not involve experimentally induced states, these effects are likely to be limited. Indeed, since all participants responded to the same set of scales in the same order, with the SDO scale always presented first, any potential order effects would have been applied equally to all respondents and therefore would not likely have introduced systematic bias in comparisons between participants. Nonetheless, future studies should randomize scale order to further minimize potential order effects. Third, while Sample 1 consisted largely of middle-class French participants with a balanced gender distribution, Samples 2 and 3 included an overrepresentation of university students, as well as a higher proportion of women. These sampling characteristics limit the generalizability of our results to the broader French population. Indeed, given that men generally score higher on SDO (Sidanius & Pratto 1999), this imbalance may have influenced the average levels observed in our data and may partly explain why our means may seem quite low. Despite observed gender differences in mean scores, measurement invariance analyses conducted on the aggregated samples (see supplemental material) confirmed that the scale exhibits an equivalent factorial structure across groups, supporting the psychometric validity of the instrument. Mean comparisons should nevertheless be interpreted in light of the sampling imbalance.

## Conclusion

Despite these limitations, this study offers a validated French adaptation of the SDO_7_ scale originally developed by Ho et al. (2015). Our findings demonstrate that the translated scale reliably captures both subdimensions of SDO and exhibits strong psychometric properties. We also propose a short version of the scale that shows acceptable reliability and validity, making it a useful alternative for time-constrained studies or experimental designs, but one that should be used with caution in cross-cultural studies. Overall, our contributions support the broader application of the SDO framework in a French-speaking context and facilitate cross-cultural comparisons with the long version of the scale in research on intergroup attitudes, ideological beliefs, and the psychological foundations of social hierarchy.

## Additional Files

The additional files for this article can be found as follows:

10.5334/irsp.1075.s1 Appendices. Appendix 1 and 2.

10.5334/irsp.1075.s2 S5. Supplementary Descriptive Items. Tables S5.1 to Table S5.4.

## Footnotes

Factor loadings for the two-factor model for the three samples are reported in the supplemental material (S4_Supplementary_FactorLoadings_Long_Version; S5_Supplementary_Descriptive_Items). We present this structure because it aligns with the current theoretical use of the scale.

We tested measurement invariance using the aggregated data from all three samples. The results supported configural, metric, and scalar invariance of the factor structure across gender, religious orientation, and sexual orientation (S6_Supplementary_Invariance_Measurement).

Additional analyses restricted to non-immigrant participants yielded equivalent results, indicating that immigrant background does not affect the outcomes (S8_Supplementary_Semipartial_Correlation).

We conducted the same analyses including only heterosexual participants. The results showed that the inclusion or exclusion of homosexual participants did not affect the outcomes (S8_Supplementary_Semipartial_Correlation).

Our mean scores ranged from 2.13 to 2.27 (*SD* = 0.85 to 0.98) for the overall SDO_7_ score, from 2.22 to 2.41 (*SD* = 0.94 to 1.03) for the D-dimension, and from 2.04 to 2.16 (*SD* = 0.95 to 1.07) for the E-dimension.
