# Supplementary material for: French Validation of the Social Dominance Orientation Scale7 (SDO7) by Ho et al. (2015): A Two-Dimensional Approach to Social Inequality Support
Source: Int Rev Soc Psychol. 2026 Apr 9;39:4. doi: 10.5334/irsp.1075 (PMC13068084; doi:10.5334/irsp.1075)
Supplement: Appendices. — Appendix 1 and 2. [file irsp-39-1075-s1.pdf]

Appendix 1

SDO_7 Scale French Version*

Indiquez dans quelle mesure vous seriez d'accord ou non avec chaque idée ci-dessous en choisissant un chiffre de 1 à 7 sur l'échelle ci-dessous. Vous pouvez travailler rapidement ; votre premier sentiment est généralement le meilleur.

1	2	3	4	5	6	7
Pas du tout d'accord	Plutôt pas d'accord	Légèrement pas d'accord	Neutre	Légèrement d'accord	Plutôt d'accord	Tout à fait d'accord

Pro-trait-Dominance

1. Certains groupes de personnes doivent être maintenus à leur place.
2. C'est sûrement une bonne chose que certains groupes soient au sommet et d'autres au bas de l'échelle.
3. Une société idéale exige que certains groupes soient au sommet et d'autres groupes soient au bas de l'échelle.
4. Certains groupes d'individus sont tout simplement inférieurs à d'autres groupes.

Con-trait Dominance

5. Les groupes du bas de l'échelle sont tout aussi méritants que les groupes du haut de l'échelle.
6. Aucun groupe ne devrait dominer dans la société.
7. Les groupes du bas de l'échelle ne devraient pas être obligés de rester à leur place.
8. La dominance de groupes est un mauvais principe.

Pro-trait Egalitarianism

9. Nous ne devrions pas insister sur l'égalité des groupes.
10. Nous ne devrions pas essayer de garantir que chaque groupe ait la même qualité de vie.
11. Il est injuste d'essayer de rendre les groupes égaux.
12. L'égalité des groupes ne doit pas être notre objectif principal.

Con-trait Egalitarianism

13. Nous devrions nous efforcer de donner à tous les groupes une chance égale de réussir.
14. Nous devons faire tout ce qui est en notre pouvoir pour égaliser les conditions de vie des différents groupes.
15. Quel que soit l'effort à fournir, nous devons nous efforcer de faire en sorte que tous les groupes aient les mêmes chances dans la vie.
16. L'égalité des groupes doit être notre idéal.

* To compute a general SDO score, items 5, 6, 7, 8, 13, 14, 15, and 16 should be reverse-coded before computing the average of all items.

To compute the SDO-D score, items 5, 6, 7, and 8 should be reverse-coded before computing the average of the first eight items.

To compute the SDO-E score, items 13, 14, 15, and 16 should be reverse-coded before computing the average of the last eight items.

Appendix 2

SDO_7 Short Scale French Version*

Descriptives, fit indices and factors loadings are available in S10_Supplementary_Short_Version.

Indiquez dans quelle mesure vous seriez d'accord ou non avec chaque idée ci-dessous en choisissant un chiffre de 1 à 7 sur l'échelle ci-dessous. Vous pouvez travailler rapidement ; votre premier sentiment est généralement le meilleur.

**1**	**2**	**3**	**4**	**5**	**6**	**7**
Pas du tout d'accord	Plutôt pas d'accord	Légèrement pas d'accord	Neutre	Légèrement d'accord	Plutôt d'accord	Tout à fait d'accord

Pro-trait-Dominance

1. C'est sûrement une bonne chose que certains groupes soient au sommet et d'autres au bas de l'échelle.
2. Une société idéale exige que certains groupes soient au sommet et d'autres groupes soient au bas de l'échelle.

Con-trait Dominance

3. Aucun groupe ne devrait dominer dans la société.
4. La dominance de groupes est un mauvais principe.

Pro-trait Egalitarianism

5. Il est injuste d'essayer de rendre les groupes égaux.
6. L'égalité des groupes ne doit pas être notre objectif principal.

Con-trait Egalitarianism

7. Nous devons faire tout ce qui est en notre pouvoir pour égaliser les conditions de vie des différents groupes.
8. L'égalité des groupes doit être notre idéal.

** To compute a general SDO score, items 3, 4, 7, and 8 should be reverse-coded before computing the average of all items.*

To compute the SDO-D score, items 3 and 4 should be reverse-coded before computing the average of the first four items.

To compute the SDO-E score, items 7 and 8 should be reverse-coded before computing the average of the last four items.
