# Supplementary material for: French Validation of the Social Dominance Orientation Scale7 (SDO7) by Ho et al. (2015): A Two-Dimensional Approach to Social Inequality Support
Source: Int Rev Soc Psychol. 2026 Apr 9;39:4. doi: 10.5334/irsp.1075 (PMC13068084; doi:10.5334/irsp.1075)
Supplement: S5. Supplementary Descriptive Items. — Tables S5.1 to Table S5.4. [file irsp-39-1075-s2.pdf]

S5. Supplementary Descriptive Items

Table S5.1: *Means, SD, skewness, min, max for each item of the 16-item ODS_7 scale for Sample 1*

ITEMS	M	SD	Skew	MIN	MAX
**1.Certains groupes de personnes doivent être maintenus à leur place.**	2.62	1.68	0.78	1	7
**2.C'est sûrement une bonne chose que certains groupes soient au sommet et d'autres au bas de l'échelle.***	2.07	1.39	1.26	1	7
**3.Une société idéale exige que certains groupes soient au sommet et d'autres groupes soient au bas de l'échelle.***	2.23	1.56	1.18	1	7
**4.Certains groupes d'individus sont tout simplement inférieurs à d'autres groupes.**	1.71	1.36	2.18	1	7
**5.Les groupes du bas de l'échelle sont tout aussi méritants que les groupes du haut de l'échelle. R**	2.26	1.59	1.19	1	7
**6.Aucun groupe ne devrait dominer dans la société. R***	2.71	1.85	0.78	1	7
**7.Les groupes du bas de l'échelle ne devraient pas être obligés de rester à leur place. R**	1.71	1.20	2.04	1	7
**8.La dominance de groupes est un mauvais principe. R***	2.49	1.60	0.87	1	7
**9.Nous ne devrions pas insister sur l'égalité des groupes.***	2.13	1.56	1.47	1	7
**10.Nous ne devrions pas essayer de garantir que chaque groupe ait la même qualité de vie.**	1.92	1.58	1.87	1	7
**11.Il est injuste d'essayer de rendre les groupes égaux.**	1.91	1.42	1.71	1	7
**12.L'égalité des groupes ne doit pas être notre objectif principal.***	2.73	1.70	0.81	1	7
**13.Nous devrions nous efforcer de donner à tous les groupes une chance égale de réussir. R**	1.45	1.01	3.07	1	7
**14.Nous devons faire tout ce qui est en notre pouvoir pour égaliser les conditions de vie des différents groupes. R***	1.97	1.35	1.32	1	7
**15.Quel que soit l'effort à fournir, nous devons nous efforcer de faire en sorte que tous les groupes aient les mêmes chances dans la vie. R**	1.71	1.16	1.74	1	6
**16.L'égalité des groupes doit être notre idéal. R***	2.53	1.66	0.90	1	7

Note: R : recoded ; * Short version ; The mean score was computed after reverse-coded items were recoded.

ITEMS	M	SD	Skew	MIN	MAX
**1.Certains groupes de personnes doivent être maintenus à leur place.**	2.90	1.61	0.48	1	7
**2.C'est sûrement une bonne chose que certains groupes soient au sommet et d'autres au bas de l'échelle.***	2.20	1.27	1.03	1	6
**3.Une société idéale exige que certains groupes soient au sommet et d'autres groupes soient au bas de l'échelle.***	2.29	1.41	0.92	1	7
**4.Certains groupes d'individus sont tout simplement inférieurs à d'autres groupes.**	1.85	1.37	1.64	1	7
**5.Les groupes du bas de l'échelle sont tout aussi méritants que les groupes du haut de l'échelle. R**	2.39	1.60	0.93	1	7
**6.Aucun groupe ne devrait dominer dans la société. R***	2.89	1.79	0.85	1	7
**7.Les groupes du bas de l'échelle ne devraient pas être obligés de rester à leur place. R**	1.99	1.33	1.86	1	7
**8.La dominance de groupes est un mauvais principe. R***	2.76	1.57	0.92	1	7
**9.Nous ne devrions pas insister sur l'égalité des groupes.***	2.08	1.39	1.11	1	7
**10.Nous ne devrions pas essayer de garantir que chaque groupe ait la même qualité de vie.**	1.99	1.56	1.60	1	7
**11.Il est injuste d'essayer de rendre les groupes égaux.**	2.04	1.51	1.27	1	7
**12.L'égalité des groupes ne doit pas être notre objectif principal.***	2.69	1.52	0.66	1	7
**13.Nous devrions nous efforcer de donner à tous les groupes une chance égale de réussir. R**	1.66	1.22	2.16	1	7
**14.Nous devons faire tout ce qui est en notre pouvoir pour égaliser les conditions de vie des différents groupes. R***	2.06	1.26	0.92	1	7
**15.Quel que soit l'effort à fournir, nous devons nous efforcer de faire en sorte que tous les groupes aient les mêmes chances dans la vie. R**	1.87	1.14	1.50	1	6
**16.L'égalité des groupes doit être notre idéal. R***	2.66	1.57	0.76	1	7

Table S5.2: Means, SD, skewness, min, max for each item of the 16-item ODS7 scale for Sample 2

Note: R : recoded ; * Short version ; The mean score was computed after reverse-coded items were recoded.

ITEMS	M	SD	Skew	MIN	MAX
**1.Certains groupes de personnes doivent être maintenus à leur place.**	2.83	1.67	0.57	1	7
**2.C'est sûrement une bonne chose que certains groupes soient au sommet et d'autres au bas de l'échelle.***	2.15	1.34	1.05	1	7
**3.Une société idéale exige que certains groupes soient au sommet et d'autres groupes soient au bas de l'échelle.***	2.30	1.50	1.05	1	7
**4.Certains groupes d'individus sont tout simplement inférieurs à d'autres groupes.**	1.81	1.38	1.84	1	7
**5.Les groupes du bas de l'échelle sont tout aussi méritants que les groupes du haut de l'échelle. R**	2.36	1.55	1.08	1	7
**6.Aucun groupe ne devrait dominer dans la société. R***	2.71	1.76	0.71	1	7
**7.Les groupes du bas de l'échelle ne devraient pas être obligés de rester à leur place. R**	1.86	1.23	1.78	1	7
**8.La dominance de groupes est un mauvais principe. R***	2.58	1.57	0.78	1	7
**9.Nous ne devrions pas insister sur l'égalité des groupes.***	2.17	1.48	1.38	1	7
**10.Nous ne devrions pas essayer de garantir que chaque groupe ait la même qualité de vie.**	1.98	1.56	1.75	1	7
**11.Il est injuste d'essayer de rendre les groupes égaux.**	2.02	1.46	1.51	1	7
**12.L'égalité des groupes ne doit pas être notre objectif principal.***	2.74	1.64	0.76	1	7
**13.Nous devrions nous efforcer de donner à tous les groupes une chance égale de réussir. R**	1.55	1.08	2.61	1	7
**14.Nous devons faire tout ce qui est en notre pouvoir pour égaliser les conditions de vie des différents groupes. R***	2.06	1.32	1.13	1	7
**15.Quel que soit l'effort à fournir, nous devons nous efforcer de faire en sorte que tous les groupes aient les mêmes chances dans la vie. R**	1.80	1.16	1.51	1	7
**16.L'égalité des groupes doit être notre idéal. R***	2.54	1.59	0.77	1	7

Table S5.3: Means, SD, skewness, min, max for each item of the 16-item ODS7 scale for Sample 3

Note: R : recoded ; * Short version ; The mean score was computed after reverse-coded items were recoded.

ITEMS	M	SD	Skew	MIN	MAX
**1.Certains groupes de personnes doivent être maintenus à leur place.**	2.83	1.67	0.57	1	7
**2.C'est sûrement une bonne chose que certains groupes soient au sommet et d'autres au bas de l'échelle.***	2.15	1.34	1.05	1	7
**3.Une société idéale exige que certains groupes soient au sommet et d'autres groupes soient au bas de l'échelle.***	2.30	1.50	1.05	1	7
**4.Certains groupes d'individus sont tout simplement inférieurs à d'autres groupes.**	1.81	1.38	1.84	1	7
**5.Les groupes du bas de l'échelle sont tout aussi méritants que les groupes du haut de l'échelle. R**	2.36	1.55	1.08	1	7
**6.Aucun groupe ne devrait dominer dans la société. R***	2.71	1.76	0.71	1	7
**7.Les groupes du bas de l'échelle ne devraient pas être obligés de rester à leur place. R**	1.86	1.23	1.78	1	7
**8.La dominance de groupes est un mauvais principe. R***	2.58	1.57	0.78	1	7
**9.Nous ne devrions pas insister sur l'égalité des groupes.***	2.17	1.48	1.38	1	7
**10.Nous ne devrions pas essayer de garantir que chaque groupe ait la même qualité de vie.**	1.98	1.56	1.75	1	7
**11.Il est injuste d'essayer de rendre les groupes égaux.**	2.02	1.46	1.51	1	7
**12.L'égalité des groupes ne doit pas être notre objectif principal.***	2.74	1.64	0.76	1	7
**13.Nous devrions nous efforcer de donner à tous les groupes une chance égale de réussir. R**	1.55	1.08	2.61	1	7
**14.Nous devons faire tout ce qui est en notre pouvoir pour égaliser les conditions de vie des différents groupes. R***	2.06	1.32	1.13	1	7
**15.Quel que soit l'effort à fournir, nous devons nous efforcer de faire en sorte que tous les groupes aient les mêmes chances dans la vie. R**	1.80	1.16	1.51	1	7
**16.L'égalité des groupes doit être notre idéal. R***	2.54	1.59	0.77	1	7

Table S5.4: Means, SD, skewness, min, max for each for each item of the 16-item ODS7 Scale for all sample

Note: R : recoded ; * Short version
